# Deep Learning Enhances Weightbearing CT Detection of Lisfranc Instability: A FIXUS-AI Ankle Insight 3D Algorithm

**DOI:** 10.7759/cureus.99658

**Published:** 2025-12-19

**Authors:** Soheil Ashkani-Esfahani, Alireza Borjali, Julian Hollander, Gregory Waryasz, Daniel Guss, Mario M Maas, Christopher W DiGiovanni, Orhun K Muratoglu, Gino M Kerkhoffs

**Affiliations:** 1 Foot and Ankle Research and Innovation Lab, Department of Orthopaedic Surgery, Mass General Brigham, Harvard Medical School, Boston, USA; 2 Department of Orthopaedic Surgery, Harris Orthopaedics Laboratory, Mass General Brigham, Harvard Medical School, Boston, USA; 3 Department of Orthopaedics and Sports Medicine, University of Amsterdam, Amsterdam, NLD; 4 Department of Orthopaedic Surgery, Mass General Brigham, Harvard Medical School, Boston, USA; 5 Department of Radiology, Amsterdam Movement Sciences, Amsterdam UMC, Location AMC, University of Amsterdam, Amsterdam, NLD; 6 Department of Orthopaedic Surgery and Sports Medicine, University of Amsterdam Academic Medical Center, Amsterdam, NLD

**Keywords:** ai and machine learning, ankle and foot, artificial intelligence in health care, deep learning artificial intelligence, expert annotation, lisfranc instability, machine learning healthcare data, machine learning in orthopaedic surgery, weightbearing computed tomography

## Abstract

Background

Recent deep learning (DL) techniques have demonstrated multiple breakthroughs in improving the detection of musculoskeletal pathologies through clinical imaging. Weightbearing CT (WBCT) has been shown to improve diagnostic accuracy in Lisfranc instability, particularly when it is subtle. The aim of the present study was to investigate the impact of applying DL algorithms on WBCT images for the diagnosis of isolated Lisfranc instability.

Methods

The WBCT scans of 280 patients were evaluated (140 cases who had isolated Lisfranc instability, 140 controls without any foot injuries). The entire data set in each group was divided into the training set, validation set, and test set with an 80:10:10 split ratio, in a random manner. Three DL models were developed: (1) a 3D convolutional neural network (3D-CNN); (2) a CNN with long short-term memory (LSTM); and (3) a differential CNN-LSTM. After training, the models’ performance was assessed by means of sensitivity, specificity, accuracy, F1-score, and the area under the receiver operating characteristic (ROC) curve.

Results

The case group included 41% males, and the control group 43%. Mean age and BMI were 35.7 and 26.6, respectively, in the case group, and 32.6 and 27.1 in controls. No significant baseline differences were found. Model 1 had an F1-score of 0.72, while Models 2 and 3 demonstrated substantially higher F1-scores of 0.92 and 0.99, respectively.

Conclusion

This study developed a DL model for 3D WBCT-based Lisfranc injury detection with excellent accuracy. The findings suggest that DL integration has the potential to improve diagnostic accuracy. Further research should focus on larger datasets and external validation.

## Introduction

The diagnosis of Lisfranc injuries can be challenging, particularly when they are subtle and limited to ligamentous injuries without associated fractures. Reports have documented a misdiagnosis rate of 13-40%, leading to delayed treatment and consequently poorer outcomes [1,2]. Among various imaging methods, weightbearing CT scan (WBCT) has been shown to be an accurate method that could provide a 3D view of the joint under physiologic weight. This enables clinicians to identify the most subtle Lisfranc instabilities with high precision [3-5]. However, interpretation of WBCT in a 3D manner is time-consuming, requires experience, and often depends on access to specialized software. Advances in technology, especially in the field of artificial intelligence (AI) and machine learning (ML), have enabled researchers and healthcare providers to improve various aspects of the quality of care, including efficiency, accuracy, and accessibility.

Recent advances in AI and ML, and more specifically deep learning (DL), have demonstrated multiple breakthroughs in the detection of musculoskeletal pathologies using clinical imaging [6-8]. Using computer-assisted image interpretation, particularly through DL algorithms, is assumed to improve not only the accuracy of diagnosis, but also the speed and costs of it. Previous reports have shown enhanced accuracy in the detection of isolated Lisfranc instabilities on weightbearing X-rays using DL algorithms [9]. Moreover, DL has been shown to improve the accuracy and speed of WBCT interpretation in the diagnosis of other foot and ankle conditions, such as isolated syndesmotic instability [5]. Therefore, the purpose of this study was to evaluate the impact of applying DL algorithms to WBCT images for the diagnosis of isolated Lisfranc instability.

## Materials and methods

The present study protocol was approved by the Institutional Review Board (IRB No. 2015P000464). Through a data inquiry sent to the Research Patient Data Registry (RPDR) system covering the years 2016-2022 across three tertiary hospitals in Boston, MA, we obtained 140 cases with intraoperatively confirmed isolated ligamentous Lisfranc instability who had undergone WBCT [5]. The study population included: 1) adults ≥18 years old, 2) patients who had undergone bilateral WBCT of the foot, and 3) individuals with no foot deformities or concomitant pathologies, including fractures or the presence of implants/hardware in the foot. We also included 140 controls who had undergone WBCT imaging but had no foot pathologies or deformities, particularly in the tarsometatarsal region.

**Figure 1:**
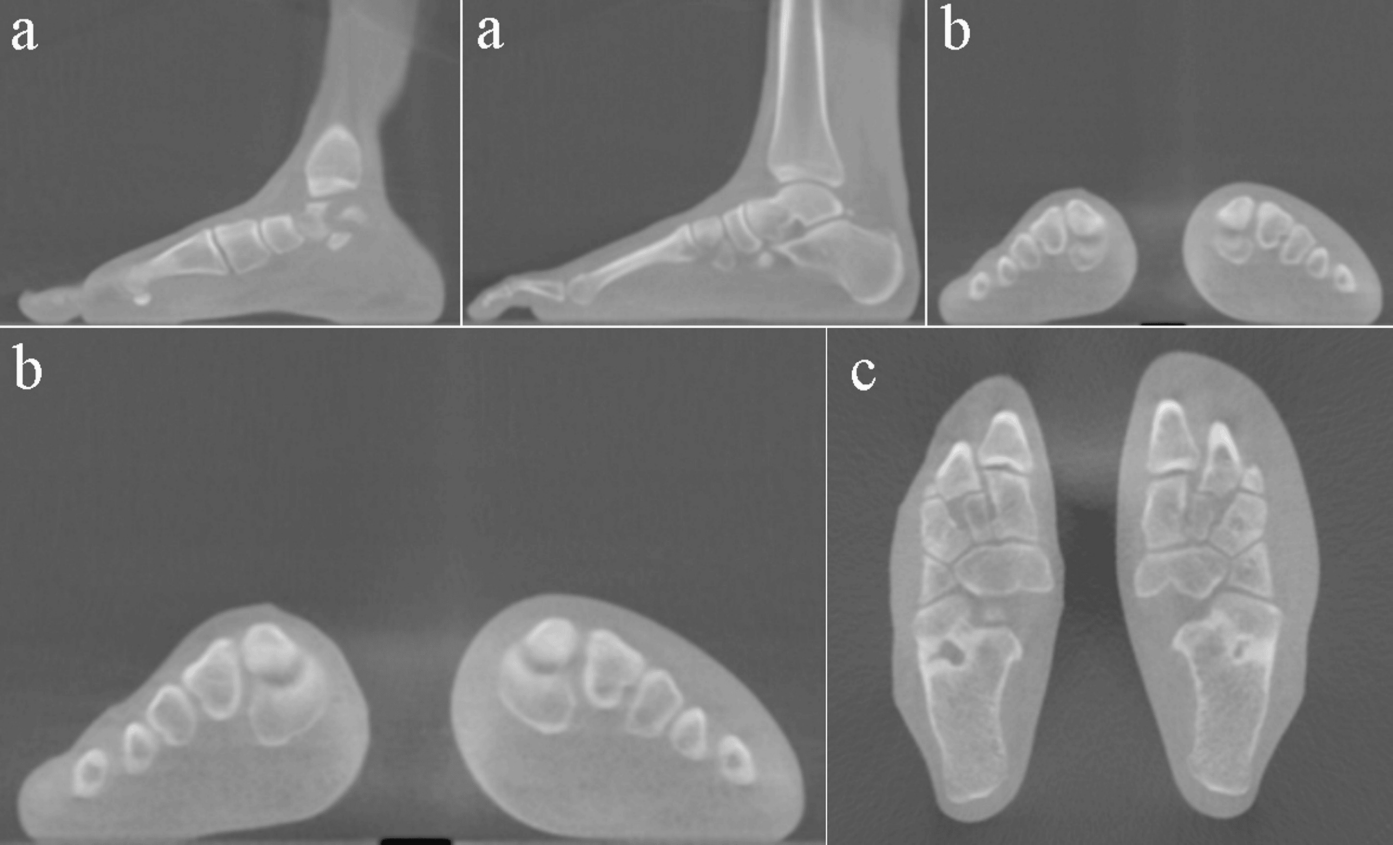
Bilateral WBCT images of the Lisfranc joint. Sagittal (a), coronal (b), and axial (c) views of the Lisfranc joint were included in the dataset to ensure capturing the joint space in a 3D manner. WBCT: weightbearing CT scan

All WBCT scans were saved in Digital Imaging and Communications in Medicine (DICOM) format. Bilateral foot WBCT images obtained in the axial, coronal, and sagittal planes with a slice thickness of 0.3 mm were used for both the case and control groups (Figure 1). We ensured that all images captured the C1-C2 and C1-M2 joint spaces in all planes. Subsequently, all CT slices were converted from DICOM to JPEG format and were rescaled to 224 × 224 pixels. These images were then normalized by dividing each pixel by 255, as the maximum pixel value. The entire dataset in each group was divided into a training set, a validation set, and a test set with an 80:10:10 split ratio in a random manner.

Deep learning models

Three different DL models were developed based on the dataset described above: (1) a 3D convolutional neural network (3D-CNN); (2) a convolutional neural network combined with long short-term memory (CNN-LSTM); and (3) a differential CNN-LSTM (DCNN-LSTM). These methods were previously used in DL-based WBCT analysis of ankle syndesmotic instability and demonstrated promise in improving diagnostic accuracy [10]. “Model 1” consisted of a 3D-CNN that was built using the full stack of WBCT scans to extract features in both temporal and spatial dimensions. Random Gaussian-distributed weights were used for initialization. “Model 2” was developed by implementing a recurrent neural network (RNN) architecture in which the output of each step depends on the previous steps, capturing sequential “memory” or extracted features. LSTM, a variation of RNN with long-term memory capabilities, can capture long-range dependencies in sequential data [11]. In Model 2, we combined a CNN (VGG-16 CNN that was pre-trained on the ImageNet dataset to extract features) with a multilayer LSTM model. Model 3, the DCNN-LSTM, utilized the same structure as Model 2; however, the inputs comprised sequential changes (ΔI) between successive slices, allowing the model to focus more efficiently on the Lisfranc joint by highlighting differences between normal and abnormal WBCT images [10]. These models were previously used on WBCT images for the diagnosis of Syndesmotic instability and showed promising results as reported by Borjali et al. [10]. All our models were trained using the Adam optimizer (initial learning rate = 0.001, beta 1 = 0.9, beta 2 = 0.999, epsilon = 1 e-8), with a batch size of 5 for 1000 epochs with early stoppage criteria. The models were implemented with TensorFlow (version r1.9) (Google Brain Team, Mountain View, CA) with Keras (version 2.2.0) (François Chollet, Google, Mountain View, CA) backend on Python (version 3.6) (Python Software Foundation, Wilmington, DE) running on a workstation with an Intel(R) Xeon(R) Gold 6128 processor, 64GB of DDR4 RAM, and an NVIDIA Quadro P5000 graphics card.

Statistical analysis

After the optimal performance was reached on the validation set, the models were tested on the test set to calculate the models’ performance. Confusion matrices were generated, followed by the calculation of sensitivity, specificity, accuracy, and F1-score. Receiver operating characteristic (ROC) curves and corresponding area under the curve (AUC) values were also generated. Dichotomous and categorical variables were reported as absolute numbers with percentages. Continuous variables were reported as mean with standard deviation (SD). Between-group differences were tested using the unpaired t-test (continuous, normally distributed data), the Mann-Whitney U test (continuous, not normally distributed), or the chi-squared test (categorical data). P < 0.05 was considered significant.

## Results

**Figure 2:**
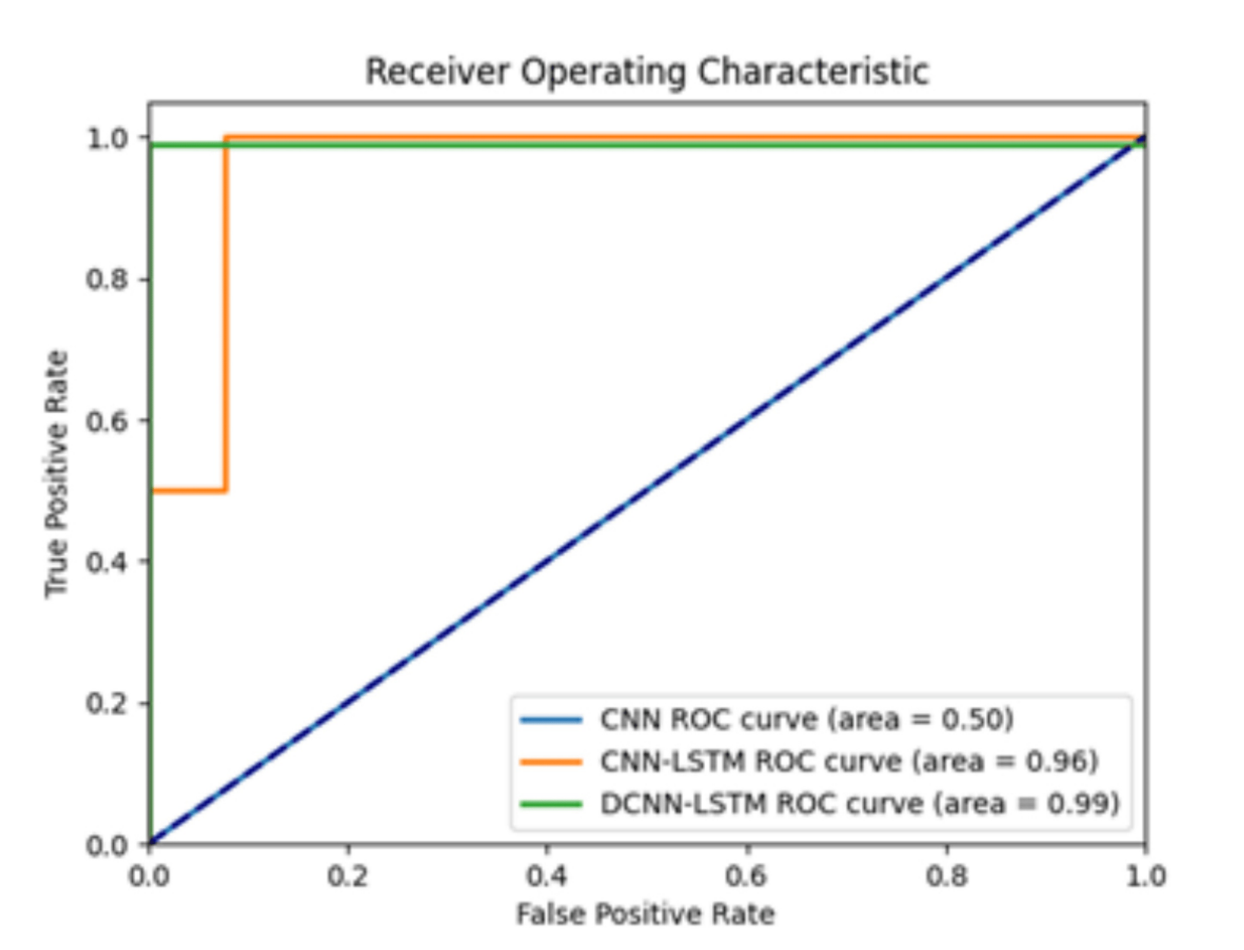
Receiver operating characteristic (ROC) curves of the three DL models. ROC curves and the area under the ROC curve of the DL models, including 3D-CNN (shown as CNN), CNN-LSTM, and differential CNN-LSTM (DCNN-LSTM). The DCNN-LSTM model showed a superior AUC, as an index for accuracy (0.99), followed by the CNN-LSTM (0.96), for the detection of Lisfranc instability. 3D-CNN: 3D convolutional neural network; AUC: area under the curve; CNN-LSTM: combined with long short-term memory; DCNN-LSTM: differential combined with long short-term memory; DL: deep learning

The demographic data of the study population are shown in Table 1. The accuracy of the 3D-CNN, CNN-LSTM, and DCNN-LSTM models was 57.1%, 91.4%, and 99.9%, respectively. The AUC of the 3D-CNN was 50.1%, while the AUC for the CNN-LSTM was 96.1% and for the DCNN-LSTM was 99.9%. The ROC curves are plotted in Figure 2. The confusion matrix for each model is available in Table 2, and the performance metrics are depicted in Table 3.

**Table 1:** Demographic characteristics of individuals in the cases and the control group. † Chi-square test, P < 0.05 considered statistically significant ‡ Student t-test, P < 0.05 considered statistically significant BMI, body mass index; SD, standard deviation

	Control group (N = 140)		Case group (N = 140)		P-value
Gender	Male	41.4% (n = 58)	Male	42.9% (n = 60)	0.21^†^
	Female	58.6% (n = 82)	Female	57.1% (n = 80)	
Age (years; mean ± SD)	35.7 ± 16.4		32.6 ± 14.2		0.23^‡^
BMI (kg/m^2^; mean ± SD)	26.6 ± 6.2		27.1 ± 5.8		0.81^‡^

**Table 2:** Confusion matrix for the three deep learning models based on the test subset of the database. Confusion matrices show the number of cases and controls that were detected correctly, incorrectly, or not detected correctly or falsely using the three DL models, including 3D-CNN, CNN-LSTM, and DCNN-LSTM. The positive and negative predictive values were calculated using the confusion matrices. 3D-CNN: 3D convolutional neural network; CNN-LSTM: combined with long short-term memory; DCNN-LSTM: differential combined with long short-term memory

		True case	True control
3D-CNN	Predicted case	13	10
	Predicted control	0	0
CNN-LSTM	-	True case	True control
	Predicted case	12	1
	Predicted control	1	9
DCNN-LSTM	-	True case	True control
	Predicted case	13	0
	Predicted control	0	10

**Table 3:** Performance metrics for three deep learning models. The sensitivity, specificity, accuracy, and F1 score (as an indicator of accuracy for DL models), were reported for the three DL models, including 3D-CNN, CNN-LSTM, and differential CNN-LSTM (DCNN-LSTM). The DCNN-LSTM showed superior performance compared to the other DL models based on these performance metrics. 3D-CNN: 3D convolutional neural network; CNN-LSTM: combined with long short-term memory; DCNN-LSTM: differential combined with long short-term memory; DL: deep learning

Model	Sensitivity	Specificity	Accuracy	F1 score
3D-CNN	1	0.10	0.57	0.72
CNN-LSTM	0.92	0.90	0.91	0.92
DCNN-LSTM	0.99	0.99	0.99	0.99

## Discussion

The present study aimed to investigate the use of DL algorithms in the interpretation of WBCT images in patients with isolated Lisfranc instability. Accordingly, three different DL-based CNN models were developed. The differential model (DCNN-LSTM) showed the best performance metrics with 99.9% AUC, while the AUCs of the CNN-LSTM and the 3D-CNN were 96.1% and 50.1%, respectively.

The superior performance of the DCNN-LSTM model might be due to the bilateral nature of our WBCT images, which enabled the algorithm to leverage side-to-side differences between the contralateral feet. Several studies have advocated for the usefulness of bilateral WBCT in the diagnosis of Lisfranc [5,12-14]. These studies have used 2D and 3D assessment methods using diastasis, area, and volume of the Lisfranc joint. The majority of these studies have shown promising accuracy when the measurements are being compared between the injured and uninjured joints, but they also report substantial interobserver variability and the time-consuming nature of 3D volume analysis. Bhimani et al. [5] included 14 patients with unilateral Lisfranc instability to assess a 3D volume measurement. They reported a sensitivity and specificity of 92.3% and 97.7% in the coronal plane, respectively. Moreover, Sripanich et al. [12] assessed WBCT images in 96 patients and concluded that WBCT is able to identify injuries of the Lisfranc joint complex, which is confirmed as they showed that the C1-M2 joint space can be accurately measured.

On radiographs, Rikken et al. [15] described an AUC ranging from 0.58 to 0.94, which is lower compared to the performance of the models in the present study. Their sensitivity and specificity also varied across measurements. For the C1-M2 joint space, they report an AUC of 0.9 with a sensitivity and specificity of 74.5% and 95.7%, respectively. When interpreting radiology, inter- and intra-rater reliability is critical to ensure valid and reproducible measurements. De Bruijn et al. examined reliability in weightbearing and non-weightbearing radiographs and found values of >0.90 for weightbearing radiographs and 0.61-0.80 for non-weightbearing radiographs. These findings further support the importance of weightbearing imaging for Lisfranc assessment [3,16,17].

The use of DL for the detection of orthopedic pathologies, specifically in the foot and ankle, has been studied by various groups of researchers [9, 18]. For example, it is concluded by Ashkani-Esfahani et al. [18] that DL can be used to detect ankle fractures faster and more precisely and assist clinicians. They reported a sensitivity and specificity of 98.7% and 98.6%, respectively. In addition, in a study that used DL to diagnose Lisfranc instability on radiographs, it was concluded that the DL algorithm outperformed human interpretation as the model only missed 5 cases (0.8%), while the radiologist (primary interpreter) missed 65 (10.2%) of the cases [9]. As with any DL system, performance varies depending on the size and quality of the dataset, annotation accuracy, image resolution, computational resources, and model architecture. Nevertheless, most researchers agree that dataset quality and size are the most influential determinants of DL performance.

The present study had several limitations. The most important limitation, consistent with many DL-based studies, is the dataset size- larger datasets generally yield more robust algorithms. However, our study represents the largest WBCT-based dataset to date for Lisfranc instability detection. Second, we pre-processed the images, reducing resolution to make the dataset computationally feasible. This may have reduced the fidelity of some features, although this trade-off was necessary given hardware constraints. Lastly, the models have not been externally validated, which limits generalizability.

## Conclusions

The present study showed that DL can improve the accuracy of WBCT in diagnosing isolated Lisfranc instability, and, through computer-assisted interpretation, may enhance the reproducibility of WBCT assessment. We reported the outcomes of DL algorithms using expert-verified labels as the ground truth. The DCNN-LSTM model demonstrated superior performance compared to the other architectures. However, future work should include model development on larger and more granular datasets to improve generalizability, as well as external validation in independent populations to ensure consistent performance across clinical settings.
